# Fishbones in the Upper Aerodigestive Tract: A Review of 24 Cases of Adult Patients

**Published:** 2017-07

**Authors:** Stanislas Ballivet-de-Régloix, Anna Crambert, Olga Maurin, Gratien Bonfort, Salome Marty, Yoann Pons

**Affiliations:** 1 *Department of ENT – Head and Neck Surgery, Military Training Hospital Percy,101, Avenue Henri Barbusse 92140, Clamart, France.*; 2 *Emergency Department, Fire Fighting Brigade of Paris, Place Jules Renard 75017, Paris, France.*

**Keywords:** Fishbone, Nasofibroscopy, Oesophagoscopy, Upper aerodigestive tract

## Abstract

**Introduction:**

We present a retrospective study series and discussion of the current literature to discuss the management of fishbones in the upper aerodigestive tract.

**Materials and Methods:**

From January 2013 to July 2016, all patients referred to our referral center because of a fishbone in the upper aerodigestive tract were analysed.

**Results:**

Of the 24 patients, 95% of them reported discomfort in the throat. It was noted that 58% of physical examinations and nasofibroscopy results were normal. Ten fishbones were found in the upper aerodigestive tract. They were removed by foreign body forceps or by endoscopy depending on the location. Foreign body-related complications were not observed. Ten patients with no identifiable fishbone had no symptoms after 48 hours. Other patients, including the 10 patients with the fishbone removed, were asymptomatic after 10 days.

**Conclusion:**

From our experience, we recommend a systematic nasofibroscopy. If it is normal, the patient is assessed at 48h. The complementary investigation by CT scan and/or oesophagoscopy must be reserved in cases of suspicion of oesophageal localization or complication. Otherwise, rigid or flexible endoscopy may be performed when laryngoscopy is unsuccessful or for the treatment of foreign bodies lodged below this area.

## Introduction

Foreign-body ingestion and aspiration are common, especially in paediatrics. Taking into account the risk of infection and digestive perforation, it often requires an endoscopic removal under conditions of maximal safety and minimal trauma, especially for short-blunt and sharp-pointed objects (1,2).

Fishbones in the upper aerodigestive tract for adult patients is less commonly explored. It poses major challenges to the laryngologist in both diagnosis and management. There is no specific recommendation concerning the management of these foreign bodies at or above the level of the cricopharyngeus (3-5). The objective of this retrospective study was to describe a case series of fishbones in the upper aerodigestive tract and develop a suitable algorithm for their management. 

## Materials and Methods

This was a retrospective study of patients referred to the Percy Military Training Hospital (Clamart, France) with fishbones in the upper aerodigestive tract from January 2013 to July 2016. Fishbones in the upper aerodigestive tract are defined as a symptomatology immediately after the ingestion of fish. Patients with foreign body aspiration and with other types of foreign body ingestion and children were excluded from the study. The hospital ethics committee exempted this study from the need for consent because it only involved retrieving data from medical records (Scientific Committee for Clinical Trials of the Percy Hospital, May 2013).

The medical files were retrospectively examined, and the following data were analysed: age, gender, mechanism of injury, functional complaint, ENT examination, nasofibroscopy, initial emergency management and medical imaging, and duration of follow-up.

*Statistical analysis:*

The statistical analysis was performed using SPSS/PC software version 10.0 (SPSS Inc. USA). The analysis was descriptive. It aimed to illustrate and explain the purpose of the discussion.

## Results

From January 2013 to July 2016, the files of 61 cases of foreign bodies in the upper airway or digestive tract were collected. Among these patients, 24 (15 men and 9 women) were analysed. The patients were, on average, 35-years-old at the time of management. After the initial consultation, all patients were assessed at 48h and 14 patients were assessed between 7 and 10 days. A history of the ingestion of fish was present in all cases, especially sea bream. All patients were examined within 48 hours.

The most frequently reported functional sign at presentation was the feeling of discomfort in the throat, more or less localized (95%). Two patients reported neck pain, especially when turning their heads, and drooling. 

The physical examination and nasofibroscopy did not reveal any foreign body in 58% of cases. Of the patients with a fishbone found via endobuccal examination or nasofibroscopy, the most common sites of impaction were the palatine tonsil (lymphoid tissue or anterior pillar) (n=3), the soft palate (n=2), the base of the tongue (n=1), the ventricular band of the larynx (n=1), and the oesophagus protruding into the pharynx (n=1). The examination of 2 patients found only local oedema (base of tongue and palatine tonsil) but not a fishbone.

CT scan was performed in 3 cases for important or persistent functional signs with normal nasofibroscopy (n=3). Fishbones were found in 2 out of 3 cases, at 2.6 cm and 4 cm below the cricopharynx (Fig.I). One patient with a normal nasofibroscopy underwent radiography of the neck and chest. It did not identify any radio-opaque foreign body. 

**Fig I:**
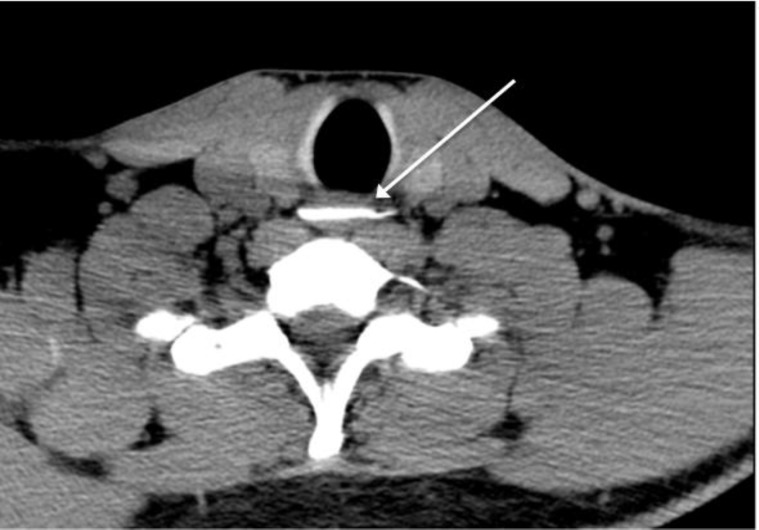
CT-scan: fishbone in the oesophagus, 4 cm below the cricopharynx (case n° 10)

Ten fishbones were found in the upper aerodigestive tract: 5 via endobuccal examina-tion, 3 via nasofibroscopy, and 2 via CT scans (Table.I). The 3 patients with foreign bodies trapped beneath the cricopharynx reported neck pain, especially when they turned their heads, and drooling. The oropharyngeal fishbones were removed by foreign body forceps after the nebulized application of lidocaine. 

 The oesophageal fishbone and the laryngeal fishbone were removed by rigid endoscopy under general anaesthesia (Table.1). No complication accountable to endoscopy has been encountered.

**Table I:** Outcomes of the patients with a fishbone

	**Functional signs**	**Physical examination / Nasofibroscopy**	**Imaging**	**Initial management**
1	Discomfort in the throat	Fishbone in the right palatine tonsil	None	Peroral removal under local anesthesia
2	Discomfort in the throat Neck pain Drooling	Nasofibroscopy : oesophagus but protuding into the pharynx	None	Removal under general anesthesia by oesophagoscopy
3	Discomfort in the throat	Fishbone in the soft palate	None	Peroral removal under local anesthesia
4	Discomfort in the throat	Fishbone in the soft palate	None	Peroral removal under local anesthesia
5	Discomfort in the throat	Fishbone in the right anterior pillar	None	Peroral removal under local anesthesia
6	Neck pain Drooling Dysphagia	Normal examination	CT-scan: fishbone in the oesophagus, 2.6 cm below the cricopharynx	Removal under general anesthesia by oesophagoscopy
7	Discomfort in throat and neck	Nasofibroscopy : fishbone in the ventricular band of larynx	None	Removal under general anesthesia by flexible endoscopy
8	Discomfort in the throat	Nasofibroscopy: fishbone in the base of tongue	None	Removal under local anesthesia by nasofibroscopy
9	Discomfort of the right palatine tonsil	Fishbone in the right palatine tonsil	None	Peroral removal under local anesthesia
10	Neck pain Drooling Dysphagia	Normal examination	CT-scan: fishbone in the oesophagus, 4 cm below the cricopharynx	Removal under general anesthesia by oesophagoscopy

Foreign body-related complications have not been observed. Ten patients with no identifiable fishbone had no symptoms after 48 hours. Other patients, including the 10 patients in whom the fishbone was removed, were asymptomatic after 10 days.

## Discussion

Fishbones in the upper aerodigestive tract for adult patients are rare in the emergency setting. Potential complications, including oesophageal perforation, mediastinitis, and cervical or mediastinal abscess, must not be neglected (6). Neck swelling, erythema, crepitus and fever must be evaluated. However, the authors did not encounter any of these factors. 

The area of discomfort often does not correlate with the site of impaction, and the main complaint is often limited to a “discomfort in the throat” or the sensation of something stuck in the neck (7).

The diagnosis is obvious if the fishbone is found by endobuccal examination or nasofibroscopy. In our series, a fishbone was found only in 10 of 24 patients, while all patients were symptomatic. What should be done when the clinical examination is normal? The clinical examination did not find any foreign body or only oedema, probably due to local trauma by fishbones. This probably means that the symptomatology persists, even though the fishbone has already been ingested after initially being planted in the throat.

Drooling is most commonly observed in patients with oesophageal foreign bodies. Associated with neck pain, this must make one think of the diagnosis and encourage complementary investigations (8).

Radiography usually identifies most true foreign objects. However, fishbones are not usually radiopaque (1,8). The clinical examination (nasofibroscopy) diagnosed only one in 3 cases of oesophageal localization. It was the CT scan that made it possible to make a diagnosis in the other 2 cases. CT scans with 3-dimensional reconstruction seem to be sufficient to diagnose the oesophageal localization of foreign body and most of the cervical and mediastinal complications (9,10).

In our series, removal with foreign body forceps under local anaesthesia and potentially under nasofibroscopic control was usually feasible. However, laryngeal and oesophageal localization was more difficult to access and required oesophagosocopy. In the literature, most ingested foreign bodies are treated with flexible endoscopes. Removal with flexible endoscopes has a high success rate and can be performed with conscious sedation in most adults, with a lower risk of perforation compared with rigid oesophagoscopy (11,12). However, rigid oesophagoscopy may be helpful for proximal foreign bodies impacted at the level of the upper oesophageal sphincter or hypopharyngeal region and may allow for the protection of the airway without an overtube, as observed in our series (13).

What should be done when the fishbone is already ingested? In the literature, the risk of a complication caused by a sharp object is up to 35%, justifying the need to endoscopically remove a sharp object that has passed into the stomach or proximal duodenum if this can be accomplished safely (14,15). Otherwise, the foreign body has to be followed with daily radiographs to document their passage (8,15). However, in our series, we did not encounter any of these complications. In addition, fishbones are not readily observed on radiography. Thus, they cannot be managed as short/blunt or sharp/pointed objects, such as chicken bones or needles. The patients should be instructed to immediately report abdominal pain, vomiting, persistent temperature elevations, haematemesis, or melena. 

## Conclusion

Fishbones in the upper aerodigestive tract are not usually responsible for complications. Patients commonly have transient symptoms at the time, such as a sensation of something stuck in the neck, dysphagia or drooling, but rarely pain. Most of the time, the foreign body is not found, and the symptoms make amends within 48 hours. When it is found, it is usually in the tonsil or soft palate. The removal of a fishbone lodged at or above the cricopharyngeus with a foreign body forceps, potentially under nasofibroscopic control, is usually feasible. From our experience, we recommend systematic nasofibroscopy. If it is normal, the patient is assessed at 48h. The complementary investigation by CT scanning and/or oesophagoscopy must be reserved in cases of the suspicion of oesophageal localization (neck pain, drooling or persistent symptoms) or complication (abscess or perforation). Otherwise, rigid or flexible endoscopy may be performed when laryngoscopy is unsuccessful or for the treatment of objects lodged below this area.

## References

[1] Holinger LD (1990). Management of sharp and penetrating foreign bodies of the upper aero-digestive tract. Ann Otol Rhinol Laryngol.

[2] Murty P, Vijendra Si, Ramakrishna S, Fahim As, Varghese P (2001). Foreign bodies in the upper aero-digestive tract. SQU Journal for Scientific Research.

[3] American society for Gastrointestinal endoscopy (2002). Guideline for the management of ingested foreign bodies. Gastrointest Endosc.

[4] Murty P, Ingle VS, Ramakrishna S, Shah FA, Varghese P (2001). Foreign bodies in the upper aero-digestive tract. J Sci Res Med.

[5] Lachaux A, Letard JC, Laugier R, Gay G, Arpurt JP, Boustière C (2007). Recommendations of the French Society of Digestive Endoscopy The ingested foreign bodies. Acta Endosc.

[6] Wilson RT, Dean PJ, Lewis M (1987). Aortoesophageal fistula due to a foreign body. Gastrointest Endosc.

[7] Connolly AA, Birchall M, Walsh-Waring GP, Moore-Gillon V (1992). Ingested foreign bodies: patient guided localization is a useful clinical tool. Clin Otolaryngol.

[8] Ikenberry SO, Jue TL, Anderson MA, Appalaneni V, Banerjee S, Ben-Menachem T (2011). Management of ingested foreign bodies and food impactions. Gastrointest Endosc.

[9] Takada M, Kashiwagi R, Sakane M, Tabata F, Kuroda Y (2000). 3D-CT diagnosis for ingested foreign bodies. Am J Emerg Med.

[10] Biswas B, Datta R (1999). retained oesophageal foreign bodies – report of three cases. Indian J Otolaryngol Head Neck Surg.

[11] Gmeiner D, von Rahden BH, Meco C, Hutter J, Oberascher G, Stein HJ (2007). Flexible versus rigid endoscopy for treatment of foreign body impaction in the esophagus. Surg Endosc.

[12] Lesur G, Vedrenne B, Heresbach D, Arpurt JP, laugier R (2009). Consensus in digestive endoscopy Materials and conditions for emergency endoscopy. Acta Endosc.

[13] Herranz-Gonzalez J, Martinez-Vidal J, Bardin-Saranderesa C, Vazquez-Barro C (1991). Esophageal foreign bodies in adults. Otolaryngol Head Neck Surg.

[14] Rosch W, Classen M (1972). Fiberendoscopic foreign body removal from the upper gastrointestinal tract. Endoscopy.

[15] Smith MT, Wong RK (2007). Foreign bodies. Gastrointest Endosc Clin N Am.

